# Multi-Locus Phylogeographic and Population Genetic Analysis of *Anolis carolinensis*: Historical Demography of a Genomic Model Species

**DOI:** 10.1371/journal.pone.0038474

**Published:** 2012-06-07

**Authors:** Marc Tollis, Gavriel Ausubel, Dhruba Ghimire, Stéphane Boissinot

**Affiliations:** 1 Biology Department, Queens College, City University of New York, Flushing, New York, United States of America; 2 Biology Program: Ecology, Evolutionary Biology and Behavior, Graduate Center, City University of New York, New York, New York, United States of America; Louisiana State University, United States of America

## Abstract

The green anole (*Anolis carolinensis*) has been widely used as an animal model in physiology and neurobiology but has recently emerged as an important genomic model. The recent sequencing of its genome has shed new light on the evolution of vertebrate genomes and on the process that govern species diversification. Surprisingly, the patterns of genetic diversity within natural populations of this widespread and abundant North American lizard remain relatively unknown. In the present study, we use 10 novel nuclear DNA sequence loci (*N* = 62 to 152) and one mitochondrial locus (*N* = 226) to delimit green anole populations and infer their historical demography. We uncovered four evolutionarily distinct and geographically restricted lineages of green anoles using phylogenetics, Bayesian clustering, and genetic distance methods. Molecular dating indicates that these lineages last shared a common ancestor ∼2 million years ago. Summary statistics and analysis of the frequency distributions of DNA polymorphisms strongly suggest range-wide expansions in population size. Using Bayesian Skyline Plots, we inferred the timing of population size expansions, which differ across lineages, and found evidence for a relatively recent and rapid westward expansion of green anoles across the Gulf Coastal Plain during the mid-Pleistocene. One surprising result is that the distribution of genetic diversity is not consistent with a latitudinal shift caused by climatic oscillations as is observed for many co-distributed taxa. This suggests that the most recent Pleistocene glacial cycles had a limited impact on the geographic distribution of the green anole at the northern limits of its range.

## Introduction

*Anolis carolinensis*, or the green anole lizard, is the first lepidosaurian reptile to have its entire genome sequenced [1]. Since its publication in 2011, the *Anolis* genome has already provided insights into our understanding of how vertebrates have diversified since the split between reptiles and mammals more than 300 million years ago (Mya) [2]. For instance, the sequence of events during the evolution of vertebrate axial skeleton segmentation as revealed by the *Anolis* genome [3] suggests that cyclically expressed patterns in the “segmentation clock” have not necessarily undergone a stepwise pattern from fish to mammals. The relative paucity of isochores in the *Anolis* genome [4] suggests that variation in GC composition is less integral to genomic structure than previously thought. In addition to these recent contributions to the field of comparative genomics, anoles as a group have been the focus of investigators in ecology and evolution for decades. The repeated convergent evolution of *Anolis* “ecomorphs” on the Greater Antilles has brought much attention to the mechanisms governing adaptive radiations (reviewed extensively in [5]). Thus, the *Anolis* genome will shed light on the genetic basis of not only morphological and ecological adaptation but the process of speciation itself [6].

While it belongs to a genus containing as many as 400 species across the tropical and semi-tropical New World, *A. carolinensis* is the only anole native to North America. Green anoles are widespread and abundant throughout the southeastern United States [7], occurring naturally in Florida (FL), Georgia (GA), North Carolina (NC), South Carolina (SC), Tennessee (TN), Alabama (AL), Mississippi (MS), Louisiana (LA), Arkansas (AR), Oklahoma (OK) and Texas (TX). Although this species has long served as a laboratory model in research fields such as physiology, neurobiology, and behavior [8], very little is known about the patterns of genetic diversity in natural populations. Molecular phylogenetic analyses have shown that *A. carolinensis* is nested within the paraphyletic *A. porcatus*, or Cuban green anole [9]; thus the ancestors of *A. carolinensis* are believed to have colonized North America via overwater dispersal from Cuba prior to the Pleistocene, where it occurs in the fossil record [10]. Morphological [11], [12] and genetic [13] analyses have concluded that there are significant differences between geographically distinct populations, including major differences between the mainland and peninsular FL. In fact, Vance (1991) proposed the subspecies status of southern FL populations (*A. c. seminolus*) on the basis of dewlap coloration and number of lamellae.

The historical processes that account for the current distribution of *A. carolinensis* remain unresolved. Eastern North America is geologically and topographically complex, and a number of common phylogeographic patterns have been reported across a wide range of co-distributed taxa [14]. Genetic discontinuities observed in both animals and plants, and notably in other squamates [15]–[18] include “latitudinal shifts” that suggest southern refugia during glacial maxima followed by northward expansion, and sharp genetic breaks associated with mountain ranges and rivers that may have acted as common barriers to dispersal.

To test hypotheses regarding historical demography, the field of phylogeography has moved towards an emphasis on incorporating as many loci as practically possible. This is because stochastic differences between the coalescent histories of gene genealogies [19] have demonstrated that a genome-wide sampling of genetic variation will better capture the signature of demographic events such as population divergences, migration, and population size changes [20]. The availability of a complete genome sequence has allowed us to test demographic hypotheses using 10 non-coding nuclear loci designed with the *Anolis* genome database and one mitochondrial locus. We have characterized and delimited evolutionary distinct lineages within this species, estimated key demographic parameters associated with its history on the continent, and tested hypotheses postulated by other comparative phylogeographic studies of southeastern North America. The results of our analyses will have important implications for future studies of *A. carolinensis*, most notably shedding light on the effects that population structure may have on the maintenance of genetic variation in a genomic model organism.

## Methods

### Ethics Statement

This study was carried out in accordance with the recommendations of the American Veterinary Medical Association (AVMA) for the euthanasia of ectotherms and every step was taken to avoid needless suffering. The following protocol was approved by the Queens College Institutional Animal Care and Use Committee (IACUC) (Animal Welfare Assurance Number: A32721-01; protocol number: 135) and was administered by the authors. After capture, animals were kept in the dark in fabric bags for a maximum of four hours and were sacrificed the same day. Euthanasia was carried out in the field via intra-abdominal injection of sodium pentobarbital at a dosage of 100 mg/kg of body weight. The euthanasia protocol approved by the University of Texas at Arlington IACUC (Animal Welfare Assurance Number: A09.012; protocol number A11.003) involved prolonged CO_2_ exposure in a closed container followed by deep freeze. The IACUC at the American Museum of Natural History does not provide welfare assurance numbers or protocol numbers, however the approved protocol ensured that animals were euthanized humanely according to methods suggested by the AVMA as well as the American Society of Ichthyologists and Herpetologists via (1) intracoelemic injection of Tricaine Methanesulfonate (MS222) with a sodium bicarbonate buffer, and (2) once the animal was confirmed as inert, a second injection of unbuffered MS222.

### Sample Collection

**Figure 1:**
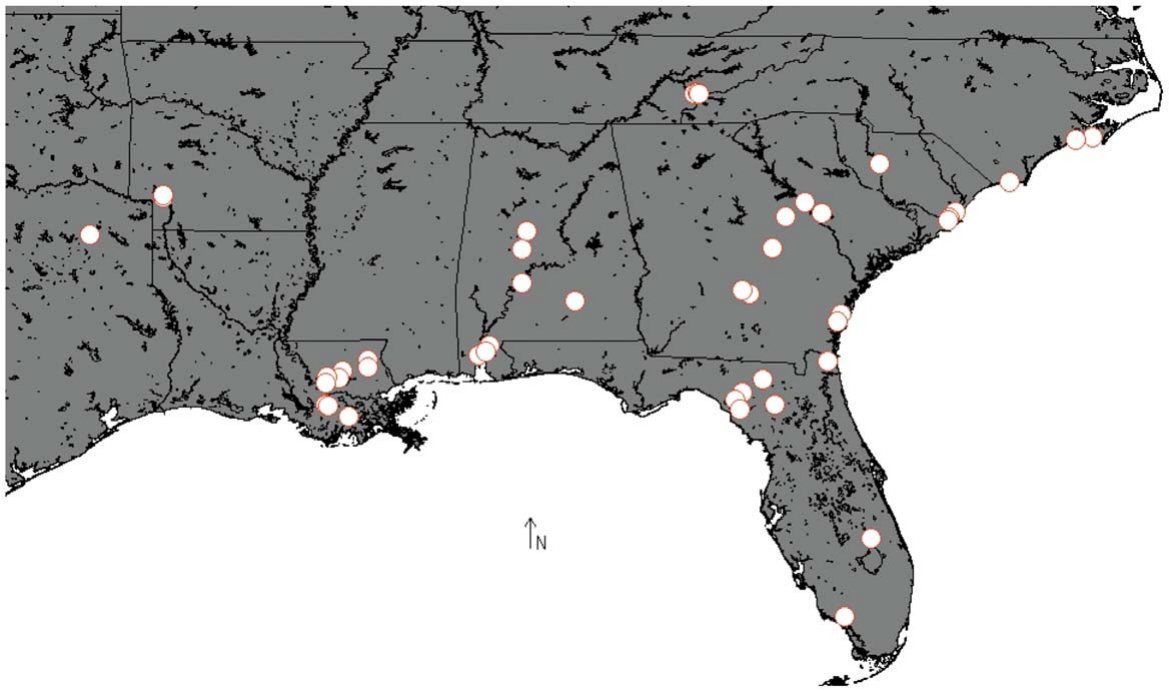
Sampling localities.

We collected 159 anoles in NC, SC, GA, FL, AL, TN, LA and AR during 2009 through 2011 and obtained their tail or liver tissues. We also obtained the tissues of nine Texan anoles from Andre Pires da Silva of the University of Texas at Arlington, and 31 blood samples from individuals collected at additional sites in SC, GA and FL given to us by Bryan Falk of the American Museum of Natural History. A map in Figure 1 shows the geographic distribution of all samples. The GPS coordinates of each collecting locality are included in Table S1. DNA was extracted from tissues via proteinase K digestion followed by purification with the Promega Wizard Genomic DNA Purification standard protocol, except for the blood samples for which we slightly modified the protocol with a smaller final elution volume to compensate for slightly lower yield.

### Marker Design, Amplification, Sequence Editing and Alignment

We designed nuclear sequence loci (nDNA) *in silico* using the UCSC Genome Browser (http://genome.ucsc.edu/) [21]. We first searched the *Anolis* genome database for introns of reasonable size (<2 Kb) for PCR amplification. An intron was chosen for further analysis if its presence was predicted by at least two of the tracks available for visualization on the browser (including NCBI RefSeq, Ensembl, non-lizard mRNAs from GenBank, and lizard ESTs). Although we selected introns for their length without respect to gene function, we recorded the putative function of each gene prediction (see Table S2). Primers were designed in the surrounding exons for Exon Primed Intron Crossing (EPIC) PCR [22] with the Primer3 program [23]. All primer pairs were used in a virtual PCR of the *Anolis* genome for specificity and single-copy confirmation. Anonymous nuclear loci were also designed using the UCSC Genome Browser. We first scanned chromosomes for gene-poor regions in order to avoid selective sweeps or background selection. Our chosen regions were used in a BLAST search [24] to screen for unannotated genes. Gene-free sequences were submitted to RepeatMasker [25] to search for repetitive elements, and a BLAT [26] of the *Anolis* genome was used for repeat detection and confirmation of single-copy status. Single copy regions lacking transposable elements, short tandem repeats, and/or single sequence repeats ranging from 200 bp to 750 bp were chosen for PCR amplification, followed by primer design using the program Primer3 and subsequent virtual PCR. All primers for PCR products used in this study are listed in Table S3. We amplified a mitochondrial region containing the nicotinamide adenine dinucleotide dehydrogenase subunit 2 (ND2) gene and downstream tryptophan and alanine tRNA genes with primers suggested by Jason Kolbe of Harvard University in 190 anoles. For each nDNA locus we amplified from a smaller geographically representative sample, resulting in 62 to 152 sequences per locus (see Table 1). PCR conditions were as follows: an initial hold for one minute at 94°C followed by 30 cycles of 30-second denaturing at 94°C, 30-second to 1 minute annealing at 54°–61°C depending on the melting temperatures of the primer pairs, and one minute extension at 72°C, with a five minute hold at 72°C before refrigeration at 4°C. All PCR products were sent to the High-Throughput Genomics Unit at the University of Washington in Seattle, WA for purification and sequencing in both forward and reverse orientations.

**Table 1:** Overiew of genetic data.

Locus	Position	bp	#ind	*s*	*k*	*π*	#haps	Hd	Fs	D
ND2	mitochondrion	1172	191	215	19.21	0.02339±0.00226	128	0.987±0.003	−34.54	−1.52
RALGAPA	chr1:24,138,759–24,139,728	970	66	7	0.98	0.00101±0.00012	8	0.513±0.048	−1.83	−0.53
HMGCS	chr2:6,161,870–6,163,157	1288	72	22	4.07	0.00317±0.00012	21	0.861±0.016	−2.85	0.07
TERT	chr4:68,148,417–68,149,503	1077	62	13	0.73	0.00067±0.00013	12	0.383±0.055	−8.27	−1.83
MON2	chr5:51,120,653–51,121,441	688	74	8	1.09	0.00138±0.00016	8	0.479±0.048	−1.31	−0.55
C1	chr2:71,967,612–71,967,955	356	72	8	1.87	0.00572±0.00027	10	0.695±0.028	−0.60	0.67
R1	chr2:54,895,768–54,896,324	557	68	12	1.61	0.00292±0.00021	9	0.664±0.36	−0.84	−0.75
Gav1	chr2:54,919,181–54,919,446	266	106	3	0.27	0.001±0.00016	4	0.255±0.038	−1.51	−0.75
Gav3	chr2:54,872,084–54,872,538	455	130	12	0.45	0.00098±0.0001	13	0.402±0.035	−12.54	−1.82
Gav4	chr2:54,927,111–54,927,321	211	152	9	0.44	0.00209±0.00024	11	0.354±035	−8.86	−1.50
Gav5	chr2:56,027,894–56,028,243	350	113	9	1.47	0.00422±0.00043	11	0.547±0.035	−1.75	−0.04

bp – fragment length in base pairs.

#ind – number of individuals sequenced at locus.

*s* – number of segregating sites.

*k* – average number of differences between sequences.

*π –* nucleotide diversity.

#haps – number of haplotypes.

Hd – haplotype diversity.

Fs – Fu’s Fs.

D – Tajima’s D (statistically significant values based on 1000 permutations in Arlequin in bold).

After Sanger sequencing, we imported chromatograms into CLC Main Workbench version 5 and Geneious v5.5 [27]. Regions of poor quality at the ends of reads were trimmed and double peaks were called using the Secondary Peak Calling option (CLC) or Find Heterozygotes plugin (Geneious) using a threshold of 50% peak height. For each sample, we assembled forward and reverse reads into contigs using a reference sequence: the virtual PCR product from the *Anolis* genome database for each nDNA locus and the ND2 region of mitochondrial sequence NC_010972 from GenBank. Putative heterozygous sites in nDNA sequence reads were assessed based on quality score. Less than 5% of all reads were unusable due to heterozygous indels, and were removed from further analyses. Each contig was edited manually and the consensus sequences were extracted and aligned using ClustalW [28] as implemented in BioEdit [29], where alignments were further edited by eye. The gametic phase of each nDNA haplotype was resolved computationally using the program PHASE 2.1 [30], with a cut off of 90% probability; phase estimation was repeated twice to assure consistent and reliable haplotype reconstruction, and the haplotypes with the highest probabilities were selected for analysis. As most phylogeographic analyses include the assumption of a lack of recombination in the data set being used, we submitted the phased haplotypes for all loci to the four-gamete test [31] as implemented in DnaSP v5 [32]. Recombination-free sequence blocks were created for the data sets in which recombination was detected by the program IMgc [33], thus rendering these loci suitable for downstream analysis. A concatenated nDNA dataset was also generated for pairwise alignments and demographic inference using SequenceMatrix [34].

### Phylogeographic Analysis

The dataset used for phylogenetic inference included 226 ND2 sequences: 190 amplified by us; the homologous region available from the *Anolis* genome sequence (NC_010972); 30 *A. carolinensis* sequences available in GenBank (accession numbers EU106323– EU106342); and three *A. porcatus* (AY654092 - AY654094), one *A. isolepis* (AY654022) and one *A. altitudinalis* (AY654023) to be used as outgroups. Phylogenies were reconstructed using the rapid bootstrap (RBS) Maximum Likelihood (ML) method in RAxML [35], a full ML analysis using MEGA 5.0 [36], and Bayesian Inference (BI) with BEAST version 1.6 [37]. For the RBS ML analysis, the sequence evolution model used was GTRCAT. Bayesian Information Criterion as implemented in MEGA indicated the most likely model of sequence evolution for this sample was HKY + Gamma + Invariant sites, and we used this model for the full ML and BI analyses, with the number of gamma categories set to 4. For the full ML analysis node support was assessed with 1000 bootstrap replicates. In order to infer the timing of diversification events in the history of *A. carolinensis* with the phylogenetic information, we first conducted a preliminary BEAST analysis (25,000,000 runs, uncalibrated with an estimated mutation rate) and calculated the average Tamura-Nei and uncorrected pairwise genetic distances between recovered clades. Assuming a mutation rate of 1.3% per million years (Myr) for this region, as observed in other small lizards [38] and used previously to date diversification events in the *Anolis* genus [9], [39], [40], we estimated the divergence time of all preliminary mtDNA clades. We used this information to calibrate the final BI tree, placing a normal prior distribution encompassing the estimated time to recent common ancestor (*t_mrca_*) of all *A. carolinensis* populations. We further calibrated our tree with knowledge from previous molecular phylogenetic analyses [9], which estimated the divergence of the *carolinensis* anole subgroup (for our purposes, the node shared by all *A. carolinensis* and *A. porcatus*) at 6Myr. The final BEAST analysis included all 226 sequences, two independent runs of MCMC length 100,000,000 with the evolutionary model and calibrations as stated above, a strict molecular clock, the known mutation rate, and a coalescent tree prior. Estimates of the posterior distributions of all parameters for each run were monitored with Tracer v1.4 [41] in order to assess convergence across separate runs, and once confirmed, separate runs were combined using LogCombiner included in the BEAST package.

We used several methods that allow us to delimit populations and infer their evolutionary history with the nDNA data. First, unrooted ML genealogies were constructed for each alignment in MEGA assuming the Tamura-Nei 1993 evolutionary model, and the topology of each genealogy was assessed in regard to congruence with each other and the mtDNA phylogeny. Multi-locus haplotypic data were entered into STRUCTURAMA 2.0 [42], which implements a Bayesian clustering algorithm to estimate the number of populations (a random variable K) using a Dirichlet prior and assigns individuals to each inferred population. We ran four independent chains of 10,000,000 generations. Haplotypes were also entered into the program *Structure 2.3.3* [43], [44], a similar clustering method that estimates the likelihoods of a range of user-set values of K. *Structure* analyses were run with 100,000 steps for burn-in followed by 100,000 generations for K values ranging from 2 to 13. Each simulation was completed five times, and results files were compressed and submitted to *Harvester* [45], which selects the most likely K value based on the delta-K criterion described by Evanno [46]. *Structure* has been shown to overestimate K (see the program documentation at http://pritch.bsd.uchicago.edu/structure.html); however, it allows the user to implement a model that includes admixture, which is a common feature of population genetic data sets. *Structure* provides estimates of the proportion of each individual’s genome derived from one of the K clusters. This differs from the STRUCTURAMA model we used, which estimates the posterior probability that each individual is a member of one of the K clusters using a no-admixture model. Therefore, similarities and differences between STRUCTURAMA and *Structure* results were interpreted such that STRUCTURAMA would recover genetic patterns on a larger geographic scale while *Structure* would be more sensitive to localized violations of Hardy-Weinberg equilibrium and therefore indicative of finer-scale population structure.

Recently, the utility of Bayesian clustering methods as the sole source of evidence for determining population structure has come under scrutiny [47], [48]. Therefore, as an additional assessment of population structure, we calculated pairwise *F_ST_* between 22 collecting localities of sample size four or greater from the concatenated dataset in Arlequin v 3.5 [49] and the resulting distance matrix was used to construct a neighbor-joining tree in MEGA. We also calculated pairwise *F_ST_* between STRUCTURAMA-inferred populations. In order to assess the degree to which the mtDNA and nDNA datasets can each explain the total genetic variation, we used Analysis of Molecular Variance (AMOVA) in Arlequin to partition groups of nDNA sequences in two ways: (1) assignment to their mtDNA clade from the phylogenetic analysis and (2) assignment to their STRUCTURAMA-inferred population. Specifically, we were most interested in the percentage of total genetic variation that is explained by differences between groups. Similar partitioning among these types of groups would suggest that both datasets recover the same phylogeographic patterns.

### Analysis of Genetic Diversity

We calculated standard diversity statistics for each locus in DnaSP including: number of polymorphic sites (s), number of haplotypes, haplotype diversity (Hd), nucleotide diversity (*π*), and average number of pairwise differences per sequence (k). Summary statistics were also calculated in Arlequin for: (1) the 22 collecting localities from which we obtained four or more individuals; (2) each inferred major mtDNA clade; and (3) each STRUCTURAMA-inferred population. We also measured the mean corrected Tamura-Nei distances within and between each mtDNA clade. The uncorrected pair-wise genetic distances per locus and the concatenated nDNA data set were measured within and between each STRUCTURAMA-inferred population. All corrected and uncorrected genetic distances were calculated in MEGA.

**Figure 2:**
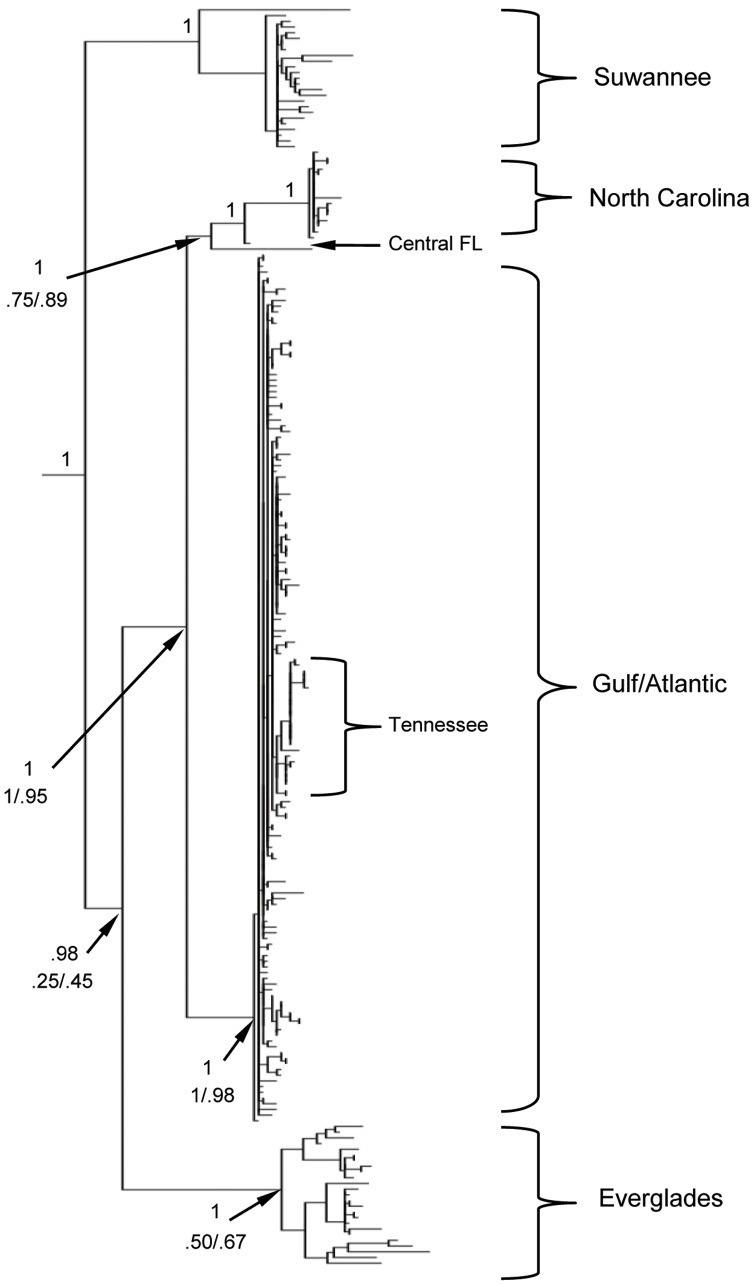
Phylogenetic reconstruction of the ND2 mitochondrial region. The most likely tree derived from the rapid bootstrap method (RBS) in RaxML is shown; the topologies of three methods (RBS, full likelihood, and Bayesian inference) were highly concordant. Posterior probabilities are given above important nodes; below nodes are the bootstrap values from RBS before the slash and from the full ML analysis after the slash. Nodes with 100% support in all three analyses are indicated with a 1. Outgroups (*A. isolepis*, *A. altitudinalis* and *A. porcatus*) are not shown. The four major clades (Suwannee, North Carolina, Gulf-Atlantic, and Everglades) are indicated with large brackets on the right. The Tennessee minor clade is shown, nested within the Gulf-Atlantic lineage. Two samples from central FL that clustered closer to the North Carolina clade are indicated with a black arrow.

### Historical Demography

In order to test for evidence of population expansion in the history of *A. carolinensis*, we calculated Tajima’s D [50] and Fu’s Fs [51] with 1000 permutations using Arlequin for each STRUCTURAMA-inferred population and major mtDNA clade and using DnaSP for each locus. For each population we also investigated the mismatch distribution of pairwise genetic differences in the concatenated nDNA using Arlequin, comparing the observed distributions to a unimodal expectation under a model of recent population expansion. The fit of the data to an expansion model is determined by a non-significant value of the raggedness index (R, [52]). Bayesian Skyline Plots [53] (BSP), which utilize the coalescent properties of gene trees to plot population size changes over time, were constructed for the mtDNA clades using BEAST. For each BSP, prior distributions for the root height of the population were notified by initial estimates from the 1.3%/My mutation rate. To incorporate stochastic differences between gene genealogies in the estimation of population parameters, as well as obtain posterior probabilities for the number of population size change events, we constructed multi-locus Extended Bayesian Skyline Plots (EBSP) [54] in BEAST for mtDNA clades. The EBSPs are informed by the known mutation rate used for ND2, and include an inheritance scalar to take into account the smaller effective population size of mtDNA versus nDNA. For BSPs and EBSPs, the lengths of the MCMC chains were set to achieve effective sampling sizes (ESS) of >200 in order to avoid autocorrelation of parameter sampling and assure proper mixing.

In cases of evidence for population expansion, we tested for directionality using a series of linear regressions with nucleotide diversity as calculated with the concatenated nDNA data from collecting localities for which we collected four or more individuals. First, *π* was plotted against latitude, in which a negative relationship could be used as evidence for southern refugia [55]. To test for east-west expansion, we plotted *π* against longitude. Recently expanded populations are not expected to show isolation by distance (IBD), as not enough time will have passed for genetic drift to differentiate geographically separated subpopulations [56]. Therefore, in the widest ranging populations, we tested for IBD by implementing the Mantel Test [57], testing the correlation between the pairwise *F_ST_* distance matrix and a geographic distance matrix generated in DIVA-GIS from the GPS points of each collecting locality. Significance was determined by 10,000 randomized permutations, and was used to accept or reject a null hypothesis of no correlation between geographic and genetic distances.

## Results

### Overview of Genetic Data

An overview of the genetic data is featured in Table 1. Excluding outgroups and sequences from GenBank, our mtDNA data set is comprised of 191 sequences of total length 1172 bp, with 215 segregating sites. Not surprisingly, the mtDNA locus exhibited higher values of diversity statistics (*k*, *π*, number of haplotypes, and Hd) than the nDNA. The number of individuals sequenced varies among nuclear loci, ranging from 62 to 74 sequences for the four introns and from 72 to 152 for the six anonymous loci (table 1). Intronic sequences ranged in length from 688 bp to 1288 bp and anonymous loci ranged from 211 bp to 557 bp. Sequences have been deposited in GenBank (accession numbers JQ857105 – JQ858187). Almost all haplotypes were reconstructed with 100% accuracy, and the very few which were estimated at <90% had no effect when removed from downstream analyses. For the nDNA loci, *π* ranged from 0.00098 to 0.00572, the number of haplotypes ranged from 4 to 21, and Hd ranged from 0.255 to 0.861. Recombination was detected in three of the 10 nDNA loci (HMGCS, C1 and Gav5), but never involved more than two events per locus, while the number of recombinant haplotypes per data set was limited to 2 or 3 individuals.

**Figure 3:**
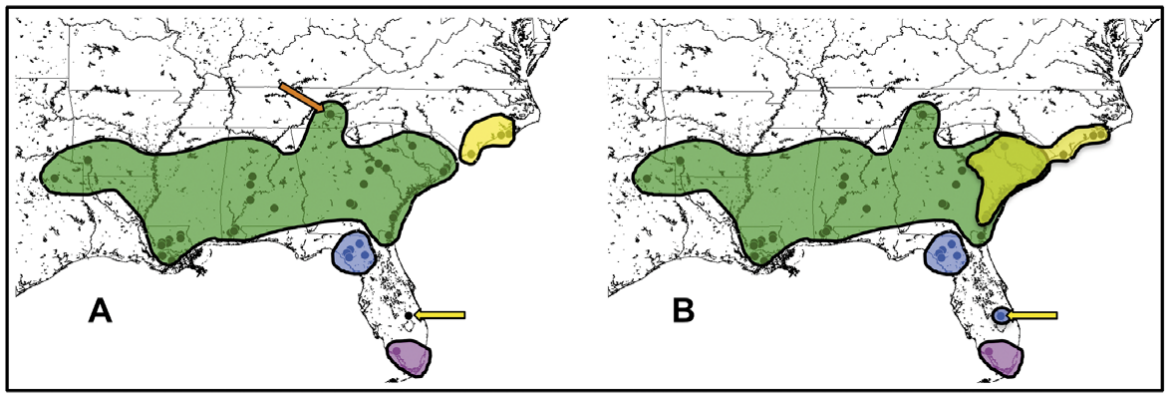
Geographic distribution of genetic populations. Colored shapes indicate the extent and boundaries of each inferred population. 3A shows the distribution of the four major mitochondrial clades: NC (yellow), Gulf/Atlantic (green), Suwannee (blue), and Everglades (magenta). The orange arrow indicates location of the Tennessee subpopulation. The yellow arrow indicates one individual in central FL that clusters with the NC clade. 3B shows the geographic distribution of the STRUCTURAMA-inferred genetic clusters. Color key is the same as 3A, except the yellow shape denotes the range of the Carolinas population inferred by nDNA versus the NC clade inferred by mtDNA. The yellow arrow points to the same individual in 3A, which clusters with the Suwannee population in the STRUCTURAMA analysis.

**Table 2:** Analysis of Molecular Variance (AMOVA).

	nDNA x mtDNA clade		nDNA x STRUCTURAMA population	
	Percentage of total variation	Fixation index	Percentage of total variation	Fixation index
Among groups	34.55	*F_CT_* = 0.34549	34.66	*F_CT_* = 0.34664
Among populations within groups	4.59	*F_SC_* = 0.07016	3.36	*F_SC_* = 0.05146
Within populations	60.86	*F_ST_* = 0.39141	61.97	*F_ST_* = 0.38027

**Table 3:** Pairwise *F_ST_* measured between STRUCTURAMA-inferred populations with nDNA.

Pairwise comparison	*F_ST_*
Gulf-Atlantic – Everglades	0.59665
Gulf-Atlantic – Suwannee	0.20249
Gulf-Atlantic – Carolinas	0.31525
Everglades – Suwannee	0.60635
Everglades – Carolinas	0.45624
Suwannee – Carolinas	0.15882

### Phylogeographic Analysis

All phylogenetic analyses yielded highly concordant topologies. The most likely tree from the RBS ML analysis of the mtDNA is shown in Figure 2, with posterior probabilities (pp) and bootstrap (bs) values shown above and below important nodes, respectively. The monophyly of *A. carolinensis* is strongly supported (1.0 bs and pp). As in the preliminary BI analysis, the final BI analysis recovered four major mtDNA clades with 100% pp. These clades are strongly correlated with geographic region (Figure 3A) and consist of (1) a lineage endemic to the Gulf coast region of FL in or around the Suwannee River drainage system (the “Suwannee” clade); (2) a group limited to anoles from the southern tip of the FL peninsula (the “Everglades” clade); (3) a NC clade and (4) a large clade including samples from all other localities ranging from the Atlantic coast of northern FL across the Gulf Coastal Plain to TX (the “Gulf-Atlantic” clade). Relationships within the Gulf-Atlantic clade could not be well resolved, with individuals from disparate geographic regions often clustering together with extremely low posterior support. One interesting well-supported minor mtDNA clade within the major Gulf-Atlantic clade consists of individuals collected from various localities on the western side of the Smoky Mountains in eastern TN. An unexpected result was the occurrence of two ND2 sequences from FL (one collected by us and one from GenBank) clustering just outside the NC clade to form a monphyletic group, and we address this below and in the Discussion section.

Given the mutation rate for the ND2 gene and the calibrations used, we estimated the age of the root of the BI tree to be 9.7 Myr (95% HPD 6.2–13.3), while the split between *A. porcatus* and *A. carolinensis* was estimated at 6.2 Myr (95% HPD 4.3–8.2). These estimates are close to estimates from past molecular phylogenetic studies [9], which propose very similar divergence dates for the *carolinensis* group and subgroup. The *t_mrca_* for all of our samples was estimated to be 2.1 Myr (95% HPD 1.3–2.9), pointing to a Late Pliocene-early Pleistocene origin for *A. carolinensis.*

We found some discordance between the mtDNA and nDNA phylogenies, due to lower variation in the nDNA and resulting multifurcations. Even so, the unrooted genealogies were useful in visualizing the concordance that did exist across most trees, showing bifurcations between FL populations and all others (Figure S1). STRUCTURAMA estimated K = 4 populations with 91% probability (2% K = 3; 7% K = 5). We named these populations Suwannee, Everglades, Gulf-Atlantic, and Carolinas, as they are largely congruent with the geographic distribution of the major mtDNA clades (Figure 3B), except for a few differences. First, STRUCTURAMA detected more gene flow between localities along the Atlantic seaboard: some individuals in the Gulf-Atlantic mtDNA clade were assigned to the STRUCTURAMA-inferred Carolinas population, which ranges from NC into coastal SC and GA. In addition, one SC and one GA collecting locality, both inland, contained individuals assigned to both the Carolinas and the Gulf-Atlantic STRUCTURAMA-inferred populations. The second difference consists of one individual collected in FL that clusters with NC anoles in the mtDNA phylogeny but was assigned to the Suwannee population in the STRUCTURAMA analysis. *Structure* simulations consistently encompassed the four STRUCTURAMA-inferred clusters. Using the delta-K method, *Structure* estimated a larger number of populations (K = 10; Delta K = 16.41) than STRUCTURAMA, the main difference being that *Structure* detected a greater degree of clustering between GA, AL, TN, LA, TX and AR in the Gulf-Atlantic STRUCTURAMA group.

**Figure 4:**
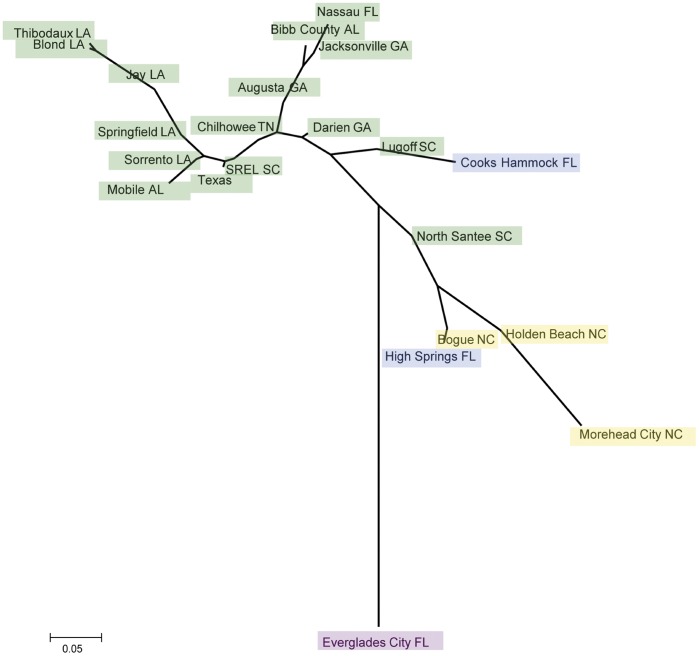
Neighbor-joining tree derived from pairwise *F_ST_* of green anole subpopulations. Colors indicate the mitochondrial clade to which individuals in the subpopulation belong: Gulf/Atlantic (green), NC (yellow), Suwannee (blue) and Everglades (magenta).

Despite these minor differences, the AMOVA partitioned the same amount of variation in the nDNA genetic data when grouped by mtDNA clade and by STRUCTURAMA-inferred group (35%, see Table 2). Therefore, both mtDNA and nDNA recover very similar geographic patterns. In both hierarchical AMOVA setups, the least amount of genetic variation existed between subpopulations within groups. This is expected if relatively little gene flow is occurring. *F_ST_* values between STRUCTURAMA-inferred populations are all significant (Table 3), suggesting strong population structure with limited gene flow between adjacent clusters. The greatest differentiation exists between the Everglades and all other populations. The NJ tree derived from the pairwise *F_ST_* distance matrix (Figure 4) recovered a pattern that is largely consistent with the cluster and phylogenetic analyses, including long branch lengths separating subpopulations from NC and SC, Southern FL, and those whose members are in the Gulf-Atlantic STRUCTURAMA-inferred group and mtDNA clade. The subpopulations whose members were assigned to the Suwannee clade/population fall in slightly disparate regions of the NJ tree, close the mid-point and with short branch lengths.

**Table 4:** Summary statistics for each mtDNA clade and STRUCTURAMA-inferred group.

	*π*		Tajima’s D		Fu’s Fs	
	mtDNA	nDNA	mtDNA	nDNA	mtDNA	nDNA
Gulf-Atlantic	0.00527	0.001422	−2.48252	−2.30077	−133.938	−26.27
Suwannee	0.02699	0.002268	−1.20207	−2.35881	−4.575	−26.04368
Everglades	0.01123	0.000853	−0.39885	−2.1162	−1.039	−20.17262
NC (Carolinas)	0.00326	0.00214	−1.58456	−0.08414	−4.576	−25.39846

### Genetic Diversity and Historical Demography

**Figure 5:**
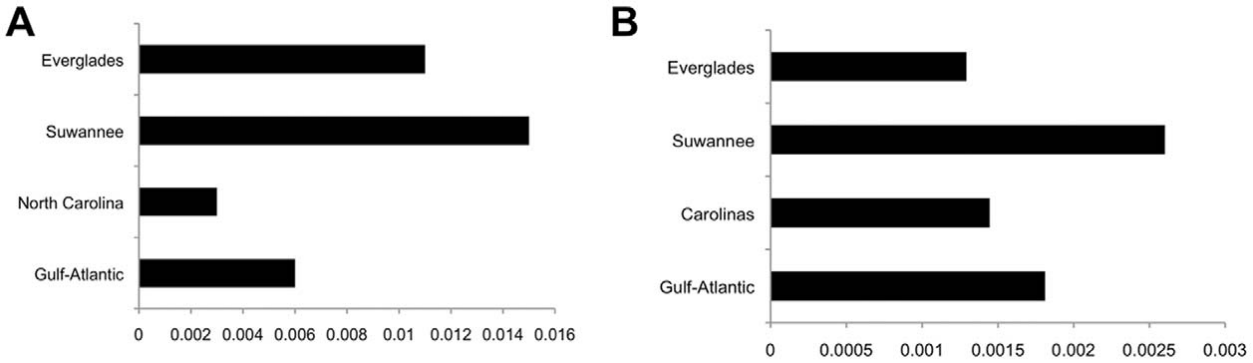
Pairwise distances within populations. A: Tamura-Nei corrected distance within each mitochondrial clade. B: Average p-distance across nine nuclear loci within each STRUCTURAMA-inferred population.

Summary statistics that were calculated per locus for each population are featured in Tables S4, S5, S6 and S7; statistics calculated for each collecting locality with the nDNA are featured in Table S8. Averaged across all loci, haplotype diversity is lowest in the Gulf-Atlantic population and highest in the FL populations. Diversity statistics for mtDNA clades and STRUCTURAMA-inferred populations (calculated from the concatenated nDNA dataset) are shown in Table 4. Nucleotide diversity is highest in the Suwannee for both data sets, and lowest in the Gulf-Atlantic and NC for the mtDNA and in the Everglades for the nDNA. Average p-distance is greatest within the Suwannee for the mtDNA (Figure 5A) and averaged across nine nuclear loci (Figure 5B). The greatest p-distance on average from all other populations is highest in the Suwannee for mtDNA and in the Everglades for nDNA; both datasets show the closest genetic distance exists between NC and the Gulf/Atlantic (Table 5).

**Table 5:** Average pair wise genetic distances within and between green anole populations.

	Gulf-Atlantic	NC (Carolinas)	Suwannee	Everglades
Gulf-Atlantic	**0.001665**	0.002566	0.002569	0.003281
	**0.006179**			
NC (Carolinas)	0.033713	**0.002361**	0.002619	0.003423
		**0.003279**		
Suwannee	0.064498	0.070281	**0.002375**	0.003526
			**0.014733**	
Everglades	0.062848	0.061362	0.075565	**0.000851**
				**0.011451**

Main Diagonal (bold): Top entry is average p-distance within the population calculated from nDNA. Bottom entry is average Tamura-Nei corrected distance within the population calculated from mtDNA. Upper diagonal: Average p-distance between two populations calculated from nDNA. Lower diagonal: Average Tamura-Nei corrected distance between two populations calculated from mtDNA.

**Figure 6:**
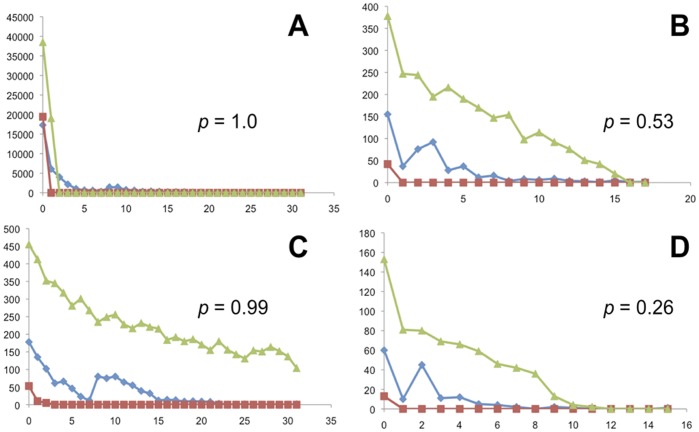
Mismatch distributions. The frequency distribution of nDNA polymorphisms within STRUCTURAMA-inferred populations calculated with the concatenated dataset in Arlequin. X-axes are in number of differences and Y-axes are in number of observations. Blue diamonds represent the observed data, green triangles and red squares represent upper and lower bounds expected under a model of expansion, respectively. P-values of the raggedness index for each analysis are given. A: Gulf-Atlantic. B: Suwannee. C: Carolinas. D: Everglades.

**Figure 7:**
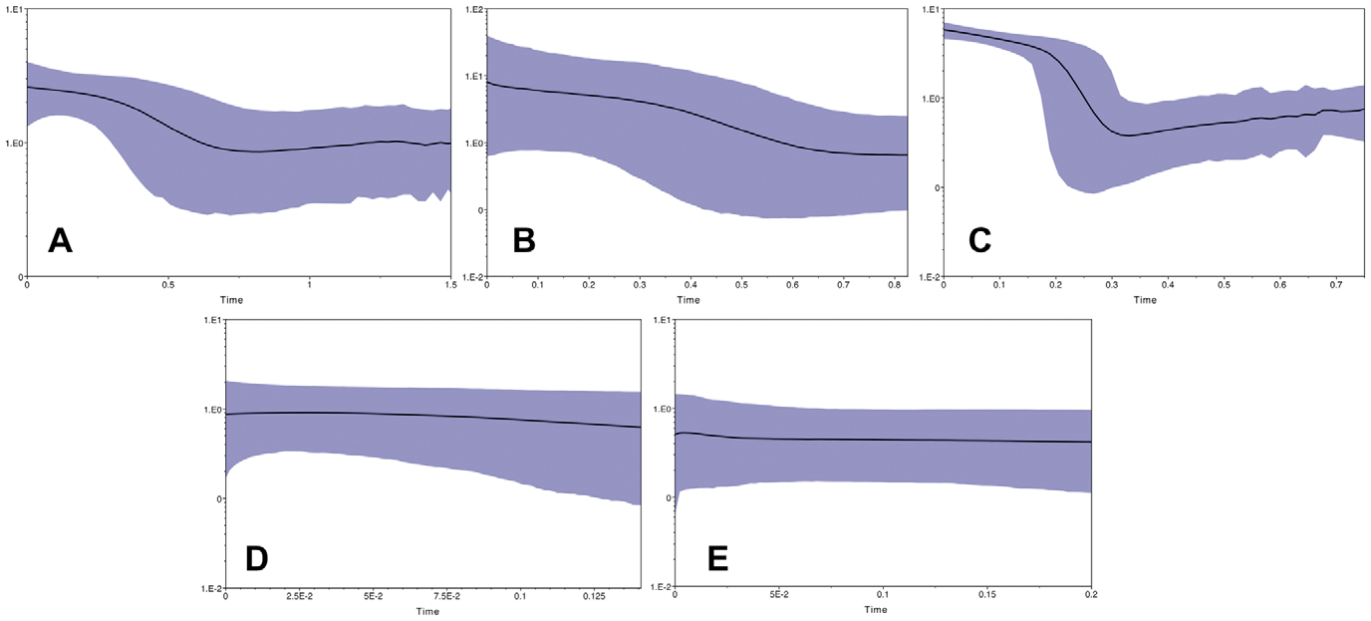
Bayesian Skyline Plots (BSPs). BSPs represent population size changes over time, inferred with mtDNA and an assumed mutation rate of 1.3% per million years. The X-axes are time in millions of years. Y-axes are mean effective population size in millions of individuals divided by generation time (for *Anolis* we assume a generation time of one year) on a log scale. Shaded areas encompass 95% highest posterior density (HPD). A: Suwannee. B: Everglades. C: Gulf/Atlantic. D: North Carolina. E: Tennessee.

**Figure 8:**
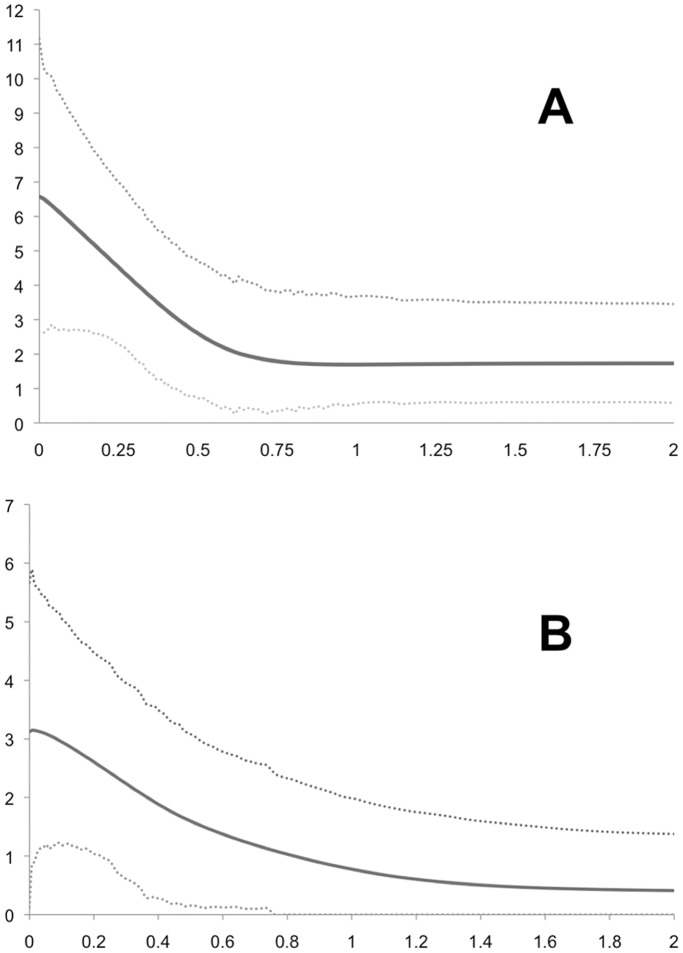
Extended Bayesian Skyline Plots (EBSPs). EBSPs represent population size changes over time in two of the mtDNA clades, inferred by mtDNA and multiple nuclear loci. Y-axes are effective population size divided by generation time. X-axes are in millions of years. A: Suwannee. B: Everglades.

**Table 6:** Estimates of effective population size obtained from the skyline plots implemented in BEAST.

	Current effective population size (millions of individuals)		
Lineage (method)	Median	Mean	95% HPD
Suwannee (BSP)	2.6	2.6	1.4–4.0
Suwannee (EBSP)	6.4	6.6	2.6–11.1
Everglades (BSP)	4	8.1	0.13–30
Everglades (EBSP)	3	3.1	0.13–5.7
Gulf-Atlantic (BSP)	5.8	5.8	4.5–7.0
North Carolina (BSP)	0.78	0.87	0.08–1.9
Tennessee (BSP)	0.41	0.5	0.03–1.2

HPD  =  highest posterior density.

Tajima’s D and Fu’s Fs were negative for most loci (Table 1), suggesting violations of neutrality due to population size expansion. Both D and Fs were also negative in all inferred populations (Table 4). The frequencies of pair-wise differences within each population (Figure 6) are consistent with what is expected under a model of population expansion: raggedness indices (R) derived from these mismatch distributions were all non-significant (*p*-values given in Figure 6). These three indicators (D, Fs, and R) all suggest that population expansion has occurred.

The BSPs indicate that the Suwannee and Everglades clades both experienced expansions ∼500–700Kya (Figure 7A and B, respectively), while the Gulf-Atlantic clade experienced a more dramatic and recent expansion ∼250Kya (Figure 7C). In contrast, the NC and TN populations have remained relatively stable during the last 150–200Ky (Figure 7D and E, respectively). Multi-locus EBSPs from the Suwannee and Everglades show highest posterior probabilities for single past population size expansions. The timing of these expansions is consistent with the single-locus BSPs (lower-bound estimates for both are between ∼0.5–1Mya) (Figure 8). Effective population size estimates from all skyline plots are given in Table 6.

A strong pattern of genetic diversity loss in northern localities was not recovered, as the regression of *π* plotted against latitude show no significant relationship (*r^2^* = 0.15; *p*-value = 0.08). The relationship between *π* and longitude was positive and significant (*r^2^* = 0.28; *p* = 0.01). When we removed the localities consisting of individuals from the Suwannee and Everglades lineages the relationship between *π* and longitude was greater (*r^2^* = 0.32; *p* = 0.01). Therefore, a stronger signal exists for an east-to-west direction of expansion. With the Mantel Test, we could not reject a null hypothesis of no correlation between geographic and genetic distances in the wide-ranging Gulf-Atlantic population (*r* = 0.18; *p* = 0.06). Thus, evidence for a model of IBD is lacking in this wide-ranging population, suggesting that the east-west expansion was rapid and relatively recent.

## Discussion

### How Many Populations?

The mtDNA phylogeny and STRUCTURAMA analysis both indicate that there are at least four distinct green anole populations. We identify these populations as (1) the Everglades in southern FL, (2) the Suwannee on the Gulf coast of FL, (3) the Gulf-Atlantic and (4) the NC clade (Carolinas for nDNA). The differences in population assignment using mtDNA and nDNA from localities in NC, SC and GA may arise in one of two ways: (1) male-biased dispersal leading to an introgression of NC nuclear haplotypes in the south and vice-versa, or (2) the retention of ancestral polymorphisms in more slowly evolving nuclear genes. Males in the *Anolis* genus are known to disperse a few hundred meters from their place of hatching [5], which is an appropriate distance considering the adjacent geography of these populations. However, more explicit modeling of this process is needed, in which the likelihoods of competing demographic hypotheses with and without migration parameters are compared. The sole anole we collected near the central Atlantic coast of FL may represent a poorly sampled mitochondrial clade endemic to that region or, more simply, an introgressed individual with a mitochondrial haplotype derived via human-mediated dispersal. More sampling is needed to address this issue.

The topology of the NJ tree based on *F_ST_* supports three of the mtDNA and STRUCTURAMA-inferred groups, with subpopulations from the putative Suwannee population occurring in disparate regions of the tree. It is important to keep in mind that this is an unrooted tree derived from the genetic distances of populations and therefore not necessarily useful for inferring evolutionary relationships. However, that these subpopulations are not separated by very long branch lengths from the midpoint of the tree may be used as further evidence for the ancestral status of the Suwannee populations (see next section). As for the *Structure* analysis, the delta-K method points to a larger K (10) than inferred by STRUCTURAMA (4). This may be because demographic histories involving population size expansions will produce an excess of low frequency polymorphisms; thus the program will use evidence from slightly divergent haplotypes to add additional clusters with minimal cost, in the process overestimating K. Still, the *Structure* results do encompass all four STRUCTURAMA groups. That STRUCTURAMA agrees so closely with the mtDNA phylogeny, and that genetic variation partitions so closely in the AMOVAs, allows us to say with confidence that four populations most accurately reflect the distribution of individuals (in our sample) across this geographic scale.

### Historical Demography of Green Anoles

It has previously been proposed that *A. carolinensis* colonized North America via overwater dispersal to FL from Cuba near the time of Plio-Pleistocene boundary [9]. Our results are consistent with this hypothesis, as the *t_mrca_* of our mtDNA dataset is estimated at ∼2 Mya. Three lines of evidence suggest that populations on the mid- and northern Gulf coast of FL represent the most persistent remnants of this colonization event: (1) the Suwannee clade is the most basal *A. carolinensis* clade in the mtDNA phylogeny; (2) genetic diversity overall is highest in this population, suggesting long-term stability; and (3) Suwannee subpopulations tend to be closer to the midpoint of the *F_ST_* NJ tree. There is a possibility that the current distribution of genetic variation represents refugial populations that were once more widespread. The genetic signatures of these populations could have been wiped out by rising sea levels and an insular history of FL during interglacial periods from the Miocene into the Pleistocene. Thus, we also cannot rule out the extinction of ancestral populations in southern FL followed by the more recent colonization of derived populations.

Once *A. carolinensis* entered the continental mainland, there were a number of geographic and topographical constraints that may have affected its dispersal patterns and thus the current distribution of individuals. Numerous studies of terrestrial fauna have described a common phylogeographic pattern showing genetic discontinuity between populations living along rivers that drain into the Atlantic Ocean and those which drain into the Gulf of Mexico [58]. Our mtDNA and nDNA datasets show that many subpopulations along the Atlantic Seaboard – particularly those south of the Charleston Harbor watershed – cluster closely to Gulf Coastal Plain populations. The oft-observed Gulf coast/Atlantic seaboard dichotomy is not absolute in *A. carolinensis*, as there appears to be extensive gene flow along the SC and GA portions where these regions overlap.

Another important discontinuity existing on either side of the Appalachian Mountains has been observed in co-distributed taxa such as salamanders [59], [60], snakes [17], [18] and turtles [61]. This break is often extended southward below the extent of the mountain chain, on either side of the Appalachicola River, which bisects the FL panhandle as it flows into the Gulf of Mexico. This pattern holds for our data but only in FL, since the Suwannee population is divergent from other Gulf coast subpopulations west of the Appalachicola (6.5% Tamura-Nei distance between them in mtDNA versus 2.4% overall). However, both mtDNA and nDNA haplotypes easily cross the hypothesized Appalachicola barrier further north, in GA and AL. More sampling around the Appalachicola river basin is needed to test its effect as an adequate barrier to dispersal, as well as the precise geographic location of genetic breaks. Unique Carolina haplotypes in both mtDNA and nDNA datasets are not found on the other side of the Appalachians in TN, despite a relatively small geographic distance. It appears that the Appalachians have acted as a barrier to dispersal further north while there also existed some form of barrier between the mainland and the Gulf coast of FL, although these need not be related phenomena.

Whether or not Pleistocene glacial cycles have had region-wide effects on co-distributed taxa has long been a subject of debate in comparative phylogeographic studies focusing on eastern North America. Southern refugia during glacial maxima followed by subsequent northward dispersal and population size expansion has been cited as an explanation for observed genetic diversity across numerous studies [14]. Our data are not consistent with this hypothesis in three ways: (1) the inferred population expansions were estimated to have pre-dated the most recent Pleistocene glacial cycles, (2) skyline plots of northern subpopulations in NC and TN show evidence of stability, and (3) there is a lack of a significant negative correlation between nucleotide diversity and latitude. While the current geographic distribution of green anole populations may reflect ancient refugia during earlier Pleistocene glacial cycles, our data suggest that more recent advances of the Laurentide Ice Sheet (∼100,000 to 10,000 years ago) have left little or no genetic signature on these populations, most notably those found at higher latitudes.

In addition to the inferred effects (or lack thereof) of late Pleistocene glaciation, the geography of genetic discontinuities in green anole populations differs from what is found in some co-distributed taxa in important ways. For instance, many of the riverine barriers that have strongly affected the cryptic fragmentation in the co-distributed common ground skink (*Scincella lateralis*) [15] have not done the same for green anoles; neither does the often observed phylogeographic break [14] at the Mississippi River hold for this species. Given the history of overwater dispersal in the *Anolis* genus [9], this is not a surprising observation, and it suggests that undetermined factors (including those inherent to the natural history of each species) have played a role in the geographic distribution of individuals in this region.

A statistically significant relationship between nucleotide diversity and longitude suggest that our data are most consistent with a hypothesis of westward expansion of *A. carolinensis* populations across the Gulf Coastal Plain during the mid- to late Pleistocene, with a minimal effect of glacial maxima during this period. Four lines of evidence suggest that anoles dispersed quite rapidly across the region: (1) mtDNA haplotypes essentially form a polytomy in the phylogenetic analysis; (2) Tajima’s D, Fu’s Fs and the mismatch distribution point to expansion; (3) a dramatic increase in population size inferred by the BSP, and (4) the lack of support for a model of isolation by distance.

We can attend to the assertion of Vance (1991) that certain southern FL populations constitute the subspecies *A. c. seminolus*, based on our analyses of the genetic data. While we have shown ample evidence for an independently evolving lineage at the southernmost reaches of the FL peninsula, we have detected an equally or even more divergent lineage endemic to the northern Gulf coast of FL. These lineages have been separate since the early to mid-Pleistocene, with minimal migration. When taken into account with previous morphological analyses that have concluded significant geographical differences, our genetic data point to significant polytypy in this species, although perhaps not as drastic as observed in ground skinks [15].

We have taken advantage of the resource provided by the *A. carolinensis* genome and devised a multi-locus study of the demographic history of this species in North America. Combining phylogenetics, clustering methods, and population genetics, we have demonstrated the existence of at least four distinct evolutionary lineages of *A. carolinensis*, the most recent common ancestor of which may predate the Pleistocene. These lineages have been characterized by population expansions since the mid-Pleistocene, and do not seem to have been affected by more recent glacial periods at mid-latitudes. The most striking phylogeographic breaks separate subpopulations from the mainland Atlantic Seaboard, the Gulf Coastal Plain, and the Gulf coast and southern portions of FL. We propose that these genetic discontinuities may result from a combination of barriers provided by the Appalachian Mountains and dispersal patterns along waterways that drain towards either the Gulf of Mexico or the Atlantic Ocean. We have also inferred a rapid mid- to late Pleistocene westward expansion of *A. carolinensis* populations across the Gulf Coastal Plain, and into habitats that have expanded the traditional niche of their more tropical evolutionary progenitors. Surely, the resources provided by the sequencing of the *Anolis* genome will provide ample opportunity to investigate the adaptive differences that exist at the molecular level for a species that ranges across such a wide variety of habitats.

## Supporting Information

Figure S1 **Unrooted ML trees for intronic sequences.** We performed phylogenetic inference using Maximum Likelihood (ML) in MEGA 5.0 with 1000 bootstrap replicates (bootstrap values not shown). Trees are unrooted due to lack of an outgroup. Circles are roughly proportional to the number of individuals present at a node, and pie charts reflect proportion of individuals at each node belonging to one of four major mitochondrial clades: Gulf-Atlantic (green), North Carolina (red), Suwannee (blue), Everlgades (magenta).
(TIF)

Table S1 **Collecting localities and GPS coordinates.**
(XLS)

Table S2 **Predicted protein products for the intronic sequences used in this study.**
(XLS)

Table S3 **Primers for PCR products used in this study.**
(XLS)

Table S4 **Summary statistics for all nuclear loci for the Gulf-Atlantic population.** #seqs – refers to phased haplotypes.
(XLS)

Table S5 **Summary statistics for all nuclear loci for the Carolinas population.**
(XLS)

Table S6 **Summary statistics for all nuclear loci for the Suwannee population.**
(XLS)

Table S7 **Summary statistics for all nuclear loci for the Everglades population.**
(XLS)

Table S8 **Summary statistics of collecting localities for which we collecting four or more individuals using the concatenated nDNA.** #seqs – refers to phased haplotypes.
(XLS)
