# Supplementary figures and images for: Multi-Locus Phylogeographic and Population Genetic Analysis of Anolis carolinensis: Historical Demography of a Genomic Model Species
Source: PLoS One. 2012 Jun 7;7(6):e38474. doi: 10.1371/journal.pone.0038474 (PMC3369884; doi:10.1371/journal.pone.0038474)

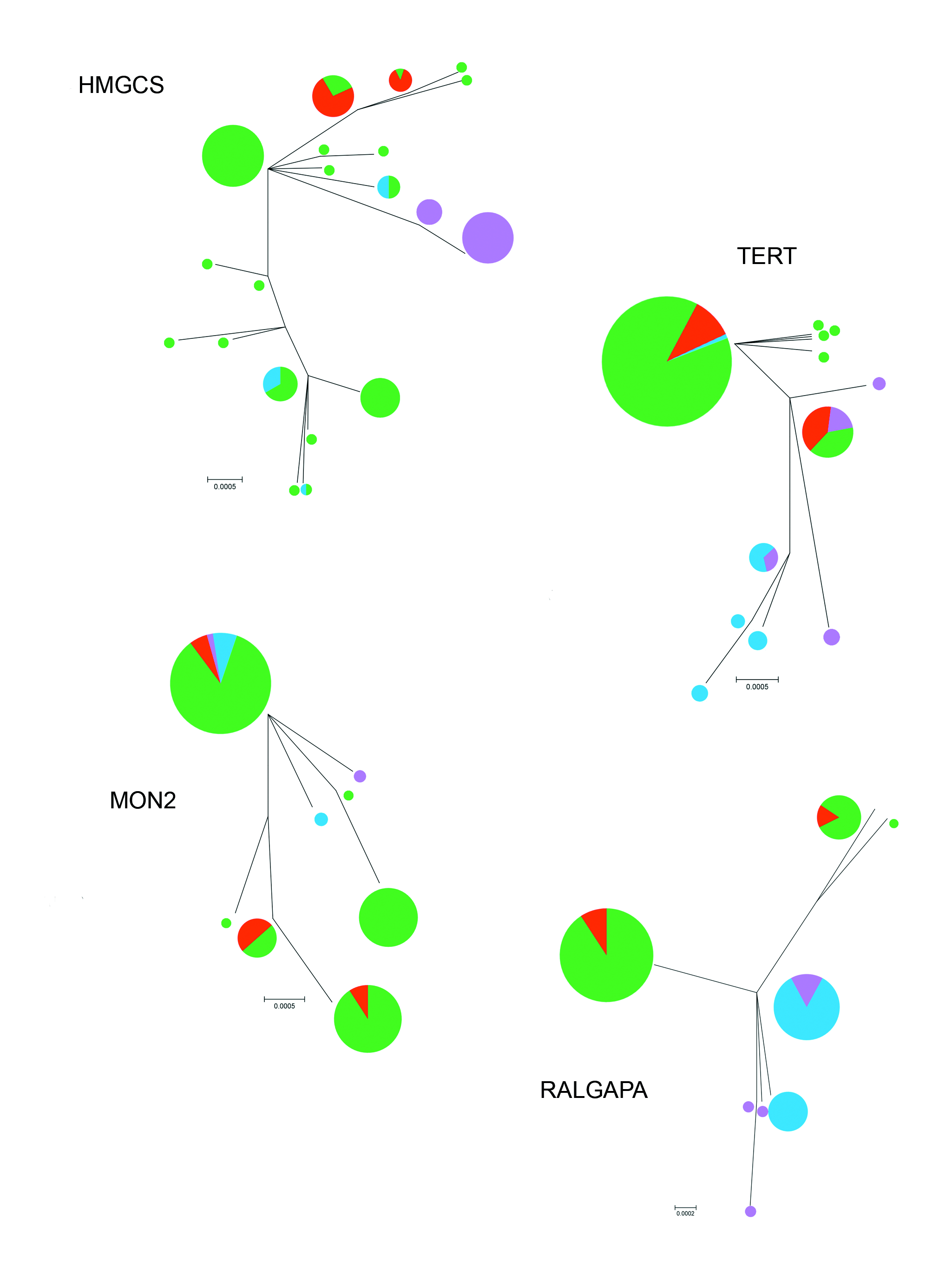

Supplement: Figure S1 — Unrooted ML trees for intronic sequences. We performed phylogenetic inference using Maximum Likelihood (ML) in MEGA 5.0 with 1000 bootstrap replicates (bootstrap values not shown). Trees are unrooted due to lack of an outgroup. Circles are roughly proportional to the number of individuals present at a node, and pie charts reflect proportion of individuals at each node belonging to one of four major mitochondrial clades: Gulf-Atlantic (green), North Carolina (red), Suwannee (blue), Everlgades (magenta). (TIF) [file pone.0038474.s001.tif]
